# Heterologous expression of a recombinant lactobacillal β-galactosidase in *Lactobacillus plantarum*: effect of different parameters on the sakacin P-based expression system

**DOI:** 10.1186/s12934-015-0214-8

**Published:** 2015-03-07

**Authors:** Tien-Thanh Nguyen, Hoang-Minh Nguyen, Barbara Geiger, Geir Mathiesen, Vincent GH Eijsink, Clemens K Peterbauer, Dietmar Haltrich, Thu-Ha Nguyen

**Affiliations:** Food Biotechnology Laboratory, Department of Food Science and Technology, BOKU University of Natural Resources and Life Sciences, Muthgasse 18, A-1190, Vienna, Austria; School of Biotechnology and Food Technology, Hanoi University of Science and Technology, 1 Dai Co Viet Street, Hanoi, Vietnam; Department of Biotechnology, Danang University of Technology, Nguyen Luong Bang 54, Danang, Vietnam; Department of Chemistry, Biotechnology and Food Science, Norwegian University of Life Sciences, P.O. Box 5003, , N-1432 Ǻs, Norway

## Abstract

**Background:**

Two overlapping genes *lacL* and *lacM* (*lacLM*) encoding for heterodimeric β-galactosidase from *Lactobacillus reuteri* were previously cloned and over-expressed in the food-grade host strain *Lactobacillus plantarum* WCFS1, using the inducible lactobacillal pSIP expression system. In this study, we analyzed different factors that affect the production of recombinant *L. reuteri* β-galactosidase.

**Results:**

Various factors related to the cultivation, i.e. culture pH, growth temperature, glucose concentration, as well as the induction conditions, including cell concentration at induction point and inducer concentration, were tested. Under optimal fermentation conditions, the maximum β-galactosidase levels obtained were 130 U/mg protein and 35–40 U/ml of fermentation broth corresponding to the formation of approximately 200 mg of recombinant protein per litre of fermentation medium. As calculated from the specific activity of the purified enzyme (190 U/mg), β-galactosidase yield amounted to roughly 70% of the total soluble intracellular protein of the host organism. It was observed that pH and substrate (glucose) concentration are the most prominent factors affecting the production of recombinant β-galactosidase.

**Conclusions:**

The over-expression of recombinant *L. reuteri* β-galactosidase in a food-grade host strain was optimized, which is of interest for applications of this enzyme in the food industry. The results provide more detailed insight into these lactobacillal expression systems and confirm the potential of the pSIP system for efficient, tightly controlled expression of enzymes and proteins in lactobacilli.

## Background

Lactic acid bacteria (LAB) have been known for a long time as important micro-organisms in the preparation and processing of a wide range of different foods, beverages and animal feed [1,2]. Being capable of rapidly converting glucose to lactic acid, LAB have been used as starter cultures in the production of a number of fermented foods in e.g. the meat and dairy industries, and have thus played an important role in human nutrition. Some lactic acid bacteria are known as producers of processing enzymes, antimicrobial peptides, or metabolites that contribute to flavor, conservation or texture of various foods. Furthermore, some LAB, in particular *Lactobacillus* spp., have been used as commercial probiotic cultures with health-promoting properties [2-4]. Based on their long-time use in food, a number of LAB carry the ‘generally recognized as safe (GRAS)’ or ‘qualified presumption of safety (QPS)’ status for human consumption.

In addition, LAB are increasingly considered as safe and attractive expression hosts and cell factories, especially for food-application purposes [2,4]. They are also attractive vehicles for *in situ* delivery of antigens or other bioactive compounds in the GI-tract [5,6]. As a consequence, a variety of constitutive or inducible gene expression and protein targeting systems have been developed for LAB [2,5,7,8]. One of the most widely used gene expression systems derived from LAB is the NIsin-Controlled gene Expression system (NICE), which is based on the autoregulatory properties and the genes involved in the synthesis of nisin, an antimicrobial peptide produced by certain strains of *Lactococcus lactis* [9]. The NICE system has been adapted to lactobacilli, but this approach has not always been straightforward or successful [10,11]. An alternative expression system, the so-called pSIP system [12], was constructed for *Lactobacillus* spp. based on the promoter and regulatory genes involved in the production of the class-II bacteriocins sakacin A [13] and sakacin P [14,15]. The production of these two bacteriocins is regulated via quorum sensing mechanisms that are based on secreted peptide pheromones with little or no bacteriocin activity [5,16,17]. The peptide pheromone (also termed inducing peptide, IP) activates a two-component regulatory system consisting of a membrane-bound histidine kinase sensing the pheromone, and an intracellular response regulator that, upon activation by the histidine kinase, induces the promoters of the operons involved in bacteriocin synthesis. In the pSIP systems, expression of the gene of interest is under control of a strong, inducible bacteriocin promoter, and gene expression is induced by external addition of the peptide pheromone. An advantage of these systems is that they are strictly regulated and lead to high production of the target protein. The applicability of these sakacin-based expression systems was shown for the over-production of enzymes such as β-glucuronidase and aminopeptidase in several *Lactobacillus* hosts [7,12].

β-Galactosidases (lactases, EC 3.2.1.23) catalyse the hydrolysis of lactose into galactose and glucose, and are important enzymes for applications in the dairy industry [18-20]. They can among others be used to produce low-lactose or lactose-free products, or prevent crystallization of lactose especially at low temperatures [21]. Moreover, β-galactosidases can catalyse transgalactosylation reactions, transferring galactosyl moieties from e.g. lactose to a suitable acceptor molecule [18]. When lactose is the primary acceptor, galacto-oligosacharides (GOS) are obtained, which are physiologically important and health-promoting prebiotic sugars [19,20,22,23]. Especially β-galactosidases obtained from known probiotic bacteria such as bifidobacteria or lactobacilli are of interest for the synthesis of these prebiotic GOS [24,25]. Nguyen *et al.* [19] screened a number of *Lactobacillus* isolates and found that one strain of *L. reuteri* exhibited high β-galactosidase activity with significant transferase activity [19]. This heterodimeric β-galactosidase of *L. reuteri* is encoded by two overlapping genes, *lacL* and *lacM.* The activity levels obtained with the wild-type strain (~2.3 kU per litre of cultivation medium, corresponding to 14 mg of β-galactosidase protein per litre) are too low to be attractive from an applied point of view. To improve these low yields, the coding regions of the two overlapping genes *lacL* and *lacM* (*lacLM)* were cloned and over-expressed in a standard expression host, *Escherichia coli* [26]. Heterologous expression in *E. coli* resulted in efficient over-expression of β-galactosidase (~110 kU/l of fermentation broth, specific activity of 55 U/mg), yet *E. coli* might not be the preferred host for food-related enzymes. As calculated from the specific activity of the purified enzyme (~180 U/mg), β-galactosidase yield amounted to roughly 30% of the total soluble intracellular protein of the host organism, hence laborious chromatographic step is required for the purification of the enzyme for further applications.

We have reported the overproduction of this enzyme in the food-grade expression host *Lactobacillus plantarum* WCSF1 [27]. The *lacLM* genes from *L. reuteri* were cloned into the expression vectors pSIP403 and pSIP409, which are based on the sakacin P operon of *L. sakei* [7,12], differing only with respect to the bacteriocin promoter that drives *lacLM* expression (P_sppA_ and P_sppQ_, respectively). This resulted in the two expression plasmids, pEH3R and pEH9R [27]. When over-expressed in the host *L. plantarum* WCFS1, cultivations of *L. plantarum* WCFS1 carrying these plasmids yielded up to ~23 kU of β-galactosidase activity, corresponding to the formation of approximately 100 mg of recombinant protein per liter of fermentation medium, and β-galactosidase levels amounted to 55% of the total intracellular protein of the host organism [27], without any optimisation of the fermentation process. The pSIP409-derived construct pEH9R was considered the better since this construct yielded lower pheromone-independent recombinant protein levels, indicative of a more strictly regulated promoter.

To further explore the (industrial) potential of the pSIP system in general and the use of lactobacilli for food-grade production of β-galactosidases in particular, we investigated the effects of various cultivation and induction conditions on gene expression. Among the factors studied were pheromone dose, timing of induction, culture pH and glucose concentration. Plasmid copy numbers during a cultivation were analyzed using reverse-transcriptase quantitative PCR. The results provide more detailed insight into these lactobacillal expression systems and show how high the expression of recombinant *L. reuteri* β-galactosidase may be achieved.

## Results

### Effect of inducer concentration, time of induction and glucose concentration

*L. plantarum* WCFS1 harbouring the plasmid pEH9R, which contains the *lacLM* genes under control of the pheromone-inducible P_sppQ_ promoter, was grown with and without pH control under various induction conditions. The concentration of the inducing pheromone (IP; a linear 19-residue peptide sometimes referred to as IP-673) was varied and the inducer was added at different growth phases of the host organism.

#### Batch cultivations without pH control

**Figure 1:**
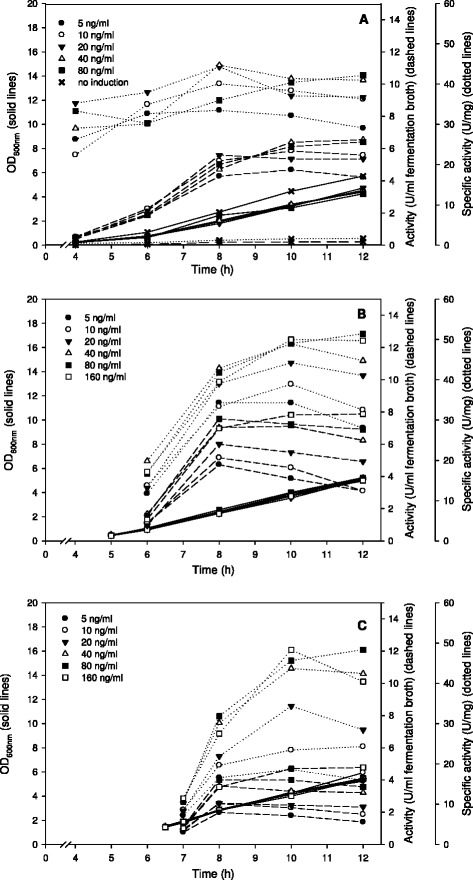
**Time course of the cultivations of** ***L. plantarum*** **overexpressing β-galactosidase from** ***L. reuteri*** **without pH control.** *L. plantarum* WCFS1 harbouring the pEH9R plasmid was grown in 50-ml cultures using MRS medium with 20 g/l glucose, at 37°C. Recombinant protein expression was induced by the addition of varying amounts of the inducing pheromone IP (ng/ml fermentation broth; see inset) at different phases of the cultivation, i.e., different OD_600_ values: immediately after inoculation of the culture **(A)**, at OD_600_ of 0.4-0.5 **(B)**, or at OD_600_ of 1.5 **(C)**. All data points represent the average value from 2 independent experiments.

Cultivations were performed without pH control at 37°C using MRS medium containing 20 g/l glucose. Despite the varying induction conditions, growth of the organism was in all cases very similar and reached an OD_600_ of ~4.5-5.0 after 12 h of cultivation (Figure 1). The volumetric activities of β-galactosidase (U per ml of fermentation broth) in induced cultures varied between 2 U/ml and 8 U/ml, and the specific activities ranged from about 20 U/mg to 50 U/mg, depending on the conditions employed. These production levels were generally reached at OD_600_ 2.0–3.0, regardless of the time of induction (immediately after inoculation, at OD_600_ of 0.4–0.5, or at OD_600_ of 1.5; Figure 1). The results show clear dose–response effects for the pheromone concentration, which level off at about 40 ng/ml. Maximum β-galactosidase levels were quite similar for cultures induced immediately after inoculation (Figure 1A) or at an OD_600_ of 0.4–0.5 (Figure 1B), but volumetric activities were clearly lower (2–4 U/ml rather than 4 – 8 U/ml) for cultures induced at OD_600_ of 1.5 (Figure 1C). These data also indicate that more pheromone is needed when induction takes place at a later growth phase. For example, induction with 20 ng/ml at OD_600_ of 0.4–0.5 maximally yielded 6 U/ml and 44 U/mg, whereas induction with 20 ng/ml at OD_600_ of 1.5 maximally yielded 2.6 U/ml and 34 U/mg. In the non-induced cultures very low enzyme activity was measured with approximately 0.2 U/ml of fermentation broth or 1.3 U/mg protein (Figure 1A). The average pH value of the fermentation media dropped from 6.5 to approximately 5.2 or 4.3 after 7 h (OD_600_ ~ 1.8-2.1) or 12 h (OD_600_ ~ 4.5-5.0) of growth, respectively.

#### Batch cultivations with pH control

**Figure 2:**
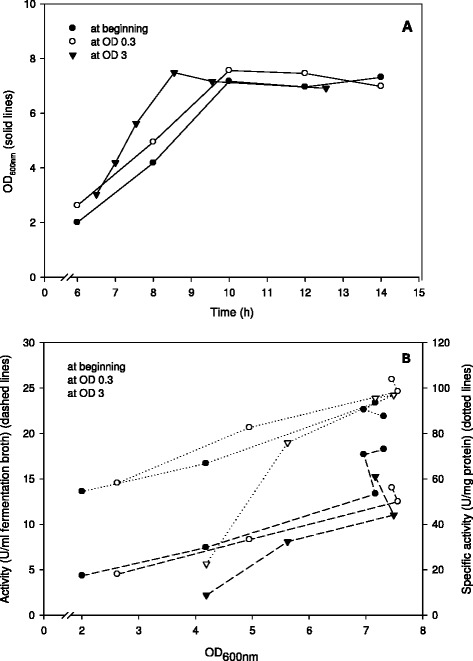
**Effect of pH control on the growth (A) and enzyme production (B) of** ***L. plantarum*** **overexpressing β-galactosidase from** ***L. reuteri*** **.** *L. plantarum* WCFS1 harbouring pEH9R was cultivated in 400-ml laboratory fermentors at 37°C using MRS medium with 20 g/l glucose and pH control at pH 6.5. Expression of β-galactosidase was induced by adding 20 ng/ml pheromone at different OD_600_: immediately after inoculation, at OD_600_ of 0.3 or at OD_600_ of 3.0. All data points represent the average value from 2 independent experiments.

In order to study the effect of the pH value on recombinant protein production when using the pSIP system, a series of cultivations was carried out where the pH was maintained at 6.5 by adding sodium hydroxide. Induction was performed using a non-saturating pheromone concentration of 20 ng/ml. The results, depicted in Figure 2A, B, show that culture pH had a strong positive effect on both growth and protein expression, and that the time of induction (immediately after inoculation, at OD_600_ of 0.3, or at OD_600_ of 3.0) hardly affected the outcome of the cultivations. OD_600_ values around 7 were reached after 10 hours of cultivation regardless of the induction time (Figure 2A) as compared to an OD_600_ of 4.5-5.0 obtained for growth without pH control (Figure 1). Accordingly, recombinant protein production was improved: β-galactosidase levels increased until the cells reached the early stationary phase to yield final volumetric activities of 15–19 U/ml, which is a 2.5–3 fold increase compared to the cultivations without pH control. Interestingly, specific β-galactosidase activities also increased about two-fold, reaching values of around 90–100 U/mg. This indicates that the improved performance of pH-controlled cultivations is not just a matter of increased cell densities.

**Figure 3:**
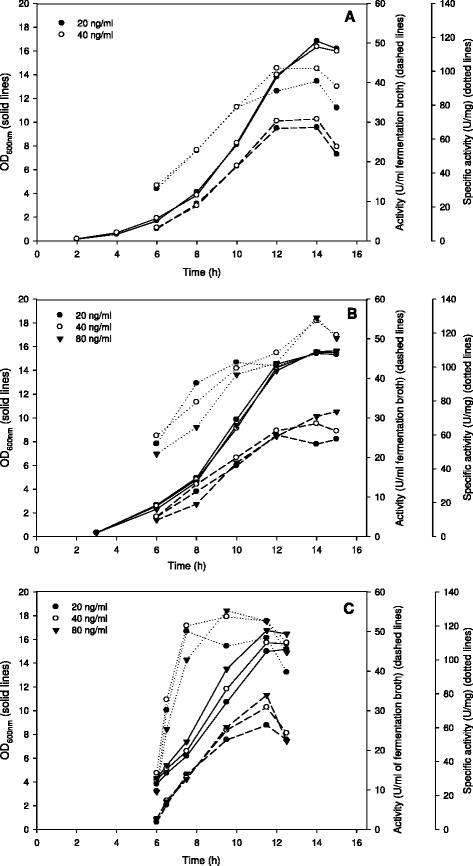
**Time course of the cultivations of** ***L. plantarum*** **overexpressing β-galactosidase from** ***L. reuteri*** **with pH control at increased glucose concentration.** *L. plantarum* WCFS1 harbouring the pEH9R plasmid was cultivated in 400-ml laboratory fermentors at 37°C using MRS medium with 40 g/l glucose and pH control at pH 6.5. Expression of β-galactosidase was induced by the addition of varying amounts of pheromone (ng/ml fermentation broth; see insert) at different OD_600_ values: immediately after inoculation **(A),** at OD_600_ of 0.3 **(B)**, or at OD_600_ of 3.0 **(C)**. All data points represent the average value from 2 independent experiments.

Subsequently, we studied the effect of varying glucose concentrations on β-galactosidase production under pH-controlled conditions (pH 6.5). Figure 3 shows that an increase of the glucose concentration from 20 g/l to 40 g/l approximately doubled the maximum OD_600_ values, which now reached 15–18. Concomitantly, the recombinant enzyme production also increased approximately two-fold; β-galactosidase levels continuously increased during the cultivation to reach a maximum of about 35 U/ml when the stationary growth phase was reached. Maximum specific activities were only slightly higher than those obtained with 20 g/l glucose, indicating that the increased volumetric yields are primarily caused by the increased cell densities. Dose–response effects for the pheromone were tested in a limited range only (20–80 ng/ml) and were generally small, as observed in other experiments for this concentration range. Comparison of the experiments displayed in Figure 3 further shows that under these conditions it may be favourable to induce somewhat later during growth since this yielded slightly higher specific activities. Higher concentrations of glucose (80, 120 g/l) were also tested, and this did not lead to a significant increase in enzyme yield even though higher cell densities were obtained (data not shown).

### Effect of antibiotic concentrations

To examine the effect of different antibiotic concentrations on recombinant enzyme production, erythromycin concentrations of 1, 5 and 10 μg/ml (final concentration in the cultivation medium) were tested using cultivation conditions similar to those described in Figure 3B. Varying the erythromycin concentrations had no significant effect on growth or recombinant protein production (data not shown). When no antibiotic was added to the culture medium, the β-galactosidase yield was much lower (approximately 2 U/ml, data not shown) than with the antibiotic added, indicating the absolute necessity to keep the selection pressure for maintaining the expression plasmid.

### Effect of temperature

Finally, we compared two different cultivation temperatures, 30°C and 37°C, with respect to growth as well as over-expression of β-galactosidase. When recombinant *L. plantarum* WCFS1 was grown in MRS medium with 40 g/l glucose and pH control at 6.5, growth and enzyme production were faster at 37°C than at 30°C. After 12 h of growth, OD_600_ values were approximately 17 and 10 for cultivations at 37°C and 30°C, respectively (data not shown). The difference in cell densities also resulted in differences in volumetric β-galactosidase activity, which ranged from 35 U/ml for 37°C to 18 U/ml for 30°C, respectively, after 12 h.

### Variation of the plasmid copy number during growth

**Figure 4:**
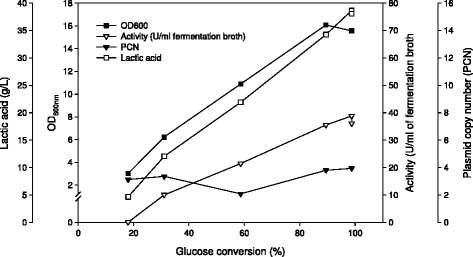
**Variation of the plasmid copy number during the cultivations of** ***L. plantarum*** **overexpressing β-galactosidase from** ***L. reuteri.*** *L. plantarum* WCFS1 harbouring the pEH9R plasmid was cultivated in 400-ml laboratory fermentors at 37°C using MRS medium with 40 g/l glucose, pH control at pH 6.5 and the cells were induced at OD ~ 3 with 80 ng/ml peptide pheromone. All data points represent the average value from 2 independent experiments.

Figure 4 shows more detailed cultivation data for an experiment run under optimal conditions. The data show that all glucose was consumed and that glucose depletion coincides with reaching maximum levels of β-galactosidase and lactic acid. To check whether the gene dose was constant during the cultivation, the plasmid copy number (PCN) was determined. The PCN was found to be at a constant level of ~4 throughout the whole exponential and stationary phase, with a slight dip in the late exponential phase.

## Discussion

Progress in genetic engineering and better understanding of various regulatory mechanisms in lactobacilli have opened the perspective of engineering these bacteria to use them as microbial cell factories and delivery vehicles for proteins. The usefulness of the pSIP vector system for high protein production has previously been shown in several studies using *L. plantarum* and *L. sakei* as host strains [7,12,27]. Most of these studies were performed in acidifying cultures in flasks, and no detailed bioreactor studies have been performed to investigate these systems in more depth. In the present study we aimed at identifying parameters that influence heterologous protein production with the pSIP vectors by using controlled cultivation conditions, and by optimizing factors such as the time and dose of induction. We used heterodimeric β-galactosidase from *L. reuteri*, encoded by the overlapping *lacLM* genes, as reporter/target protein in the optimization studies as the highest expression levels were obtained in a laboratory cultivation of *L. plantarum* WCFS 1 harbouring the plasmids containing these genes [27].

As expected the β-galactosidase yield was very low in non-induced cultures, while specific activities of up to 130 U/mg were found under appropriate induction and growing conditions, giving typical induction factors (ratio of specific activity under induced and non-induced conditions) of more than 100. This illustrates the tight control of the system, in agreement with previous studies of the pSIP expression system [7,12]. It should be noted that background β-galactosidase activity caused by expression of the chromosomal *lacLM* genes of *L. plantarum* are negligible (<0.1 U/mg) when the strain is grown on glucose. Hence, the activities reported in this paper can be considered as originating exclusively from heterologous expression of the vector-based *lacLM* genes. We observed a clear dose–response effect up to IP concentrations of ~40 ng/ml (Figures 1 and 3), and none of the tested IP concentrations had inhibitory effects on growth of *L. plantarum* as is evident from the almost identical growth curves depicted in Figures 1 and 3. Apart from showing that the IP itself is not inhibitory up to the highest tested concentration of 160 ng/ml, this also shows that the cells are capable of handling the high amounts of heterologous protein very well.

The yield of the recombinant protein was affected by the induction time point (growth phase), but only in the experiments without pH control. In these experiments, induction at high optical density (OD_600_ ~ 1.5) resulted in lower volumetric activities than induction at low OD_600_, and higher pheromone concentrations were needed to reach maximum expression levels (Figure 1). The absence of this effect in cultures with pH control (Figures 2 and 3) indicates that the pH at the time of induction has influence on the induction efficiency, as has been suggested previously [14].

**Figure 5:**
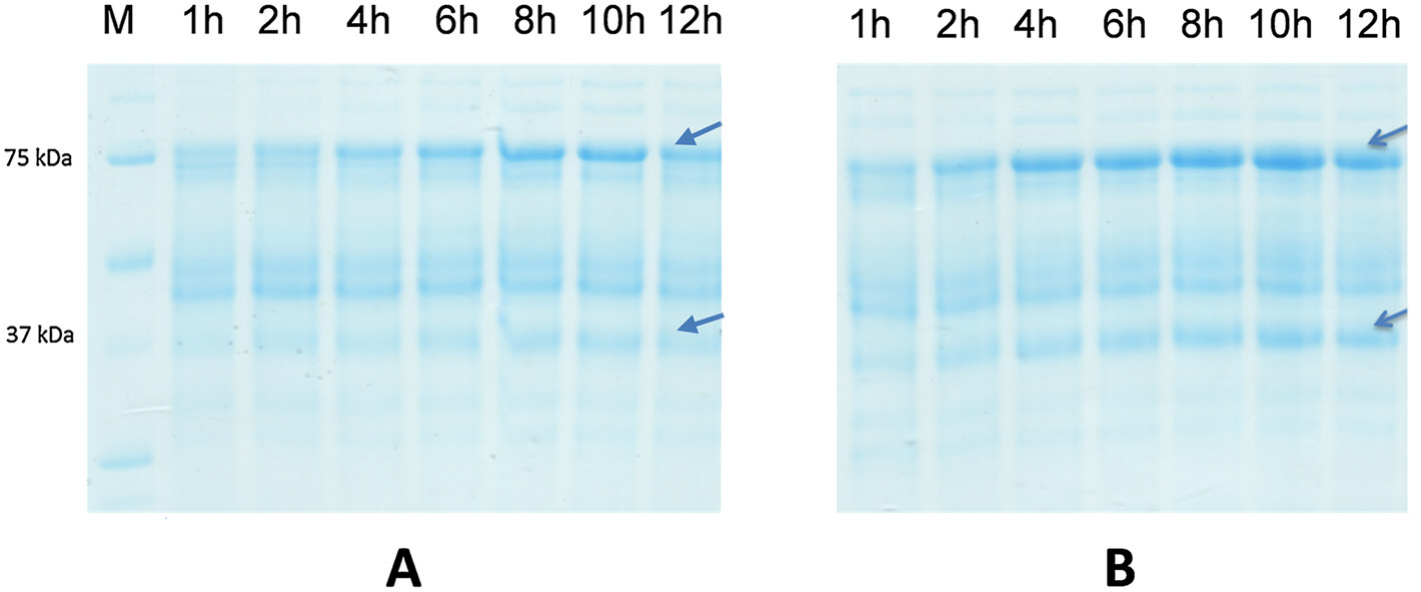
**SDS-PAGE analysis of cell free extract of crude** ***L. plantarum*** **overexpressing β-galactosidase from** ***L. reuteri*** **from the cultivations without pH control (A) and with pH control at pH 6.5 (B).** *L. plantarum* WCFS1 harbouring pEH9R was cultivated in 400-ml laboratory fermentors at 37°C using MRS medium with 20 g/l glucose, and samples were taken at different time points. The arrows indicate the LacL and LacM subunits of the recombinant β-galactosidase. M denotes the Precision protein ladder (Biorad, CA, USA).

Maintaining the pH at a set value of 6.5 was clearly beneficial for β-galactosidase yields, both in terms of the volumetric and the specific β-galactosidase activities. This indicates that the decrease in pH during a non-controlled cultivation has a negative effect of the production of β-galactosidase. As expected the constant pH of 6.5 led to increased cell densities. However, this increase in biomass cannot solely explain the higher yields of recombinant protein, as indicated by the considerably higher specific activities that were obtained. One possible beneficial effect of the constant pH could be higher effectiveness of the induction process, as mentioned above. The difference in specific activities between pH controlled and non-controlled fermentations was further confirmed by SDS-PAGE analysis of cell free crude extract obtained from these cultivations (Figure 5), with the bands for the recombinant β-galactosidase being more prominent for the samples obtained with pH control.

Even higher enzyme yields were obtained when the initial glucose concentration was increased, with maximum β-galactosidase levels being reached at 40 g/l glucose. This showed that glucose is the limiting factor in standard MRS medium. The maximum β-galactosidase levels obtained in these experiments (130 U/mg protein and 35–40 U/ml of fermentation broth) correspond to approximately 180 mg of recombinant protein produced per litre of fermentation medium as calculated from the specific activity of purified enzyme of 190 U/mg, which corresponds to roughly 70% of the total soluble intracellular protein being recombinant β-galactosidase. This is one of the highest expression levels obtained with gene expression systems in lactic acid bacteria to date [9].

It was previously reported that temperature can affect bacteriocin-related quorum sensing mechanisms in lactobacilli [28], and thus perhaps also expression levels for the pSIP system. We did, however, not observe significant differences in yield when comparing results at identical cell densities in pH-controlled cultivations performed at these two temperatures.

The expression system functioned well at antibiotic concentrations down to 1 μg/ml but the experiments also showed that the system does not work without antibiotics at all. Recent studies on segregational stability of pEH9R in *L. plantarum* WCFS1 showed that absence of erythromycin leads to a decrease in the number of cells harbouring plasmid pEH9R [29]. This indicates the absolute necessity to maintain strict selection pressure on the pSIP expression system during the cultivation. Because of the modular structure of the SIP system [5,12] it is easy to exchange selection markers e.g. with complementation markers such as the alanine racemase gene (*alr*) [30,31] or the lactose carrier *LacF* [32], which makes the addition of antibiotics redundant. We recently developed pSIP variants based on *alr* as selection marker, and tests done so far indicate that these vectors perform equally well as the original *ery*-based vectors in terms of protein expression and stability [29].

The pEH9R plasmid was found to be present in low copy numbers (approximately 2–4, depending on the growth phase), and this is in accordance with the findings that the 256_rep_ replicon is a low-copy-number replicon in *Lactobacillus* [33]. A decrease in the PCN was observed after approx. 10 h of cultivation, later during the exponential growth phase. A possible explanation for this could be that because of the fast duplication of the cells during this phase of rapid growth, the cellular machinery cannot provide the daughter cell with a sufficient number of the plasmids. When the growth rate subsequently decreased again, the PCN increased to the original value of approximately 4, and then stayed constant also during the stationary phase. It is interesting to note that the exceptionally high levels of recombinant protein, amounting to about 70% of total intracellular protein, were achieved with a gene dose not higher than approximately four.

## Conclusion

We here described the optimization in terms of growth and induction conditions for the over-expression of a recombinant β-galactosidase using a pSIP409-based expression vector in *Lactobacillus plantarum* WCFS1. The highest β-galactosidase levels obtained were 130 U/mg protein and 35–40 U/ml of fermentation broth, which corresponds to roughly 70% of the total soluble intracellular protein being recombinant β-galactosidase.

## Materials and methods

### Bacterial strains and media; fermentations

*L. plantarum* WCFS1 [34] harbouring pEH9R [27], which contains the overlapping genes (*lacLM*) coding for β-galactosidase of *L. reuteri* L103 [26], was grown at 37°C in 5 ml of MRS containing 5 μg/ml erythromycin for 16–18 h. Such overnight cultures were used as inoculum for subsequent cultivations.

For batch fermentations without pH control, 1% (v/v) of inoculum was added to 50 ml of medium, and the cultures were grown in 50 ml tightly closed bottles at 37°C. Batch fermentations with pH control were carried out in 400 ml medium in HT-Multifors fermentors (Infors HT, Switzerland); also in this case cultures were inoculated with 1% (v/v) of an overnight preculture. The pH was controlled at pH 6.5 using sodium hydroxide when stated, and agitation was set at 200 rpm. Glucose concentrations in the MRS medium were varied as indicated. Gene expression was induced by adding varying levels of the synthetic pheromone IP-673 at different time points. IP-673 is a 19-amino acid peptide synthesised commercially according to the sequence of the original pheromone from *Lactobacillus sakei* LTH673 [35].

Samples were taken periodically to measure optical density at 600 nm, β-galactosidase activity and the PCN. For β-galactosidase measurements, cells from 1 ml of culture were harvested by centrifugation at 16000 *g* for 3 min, cell pellets were re-suspended in sodium phosphate buffer (buffer P) [22], and then disrupted by sonication (Bandelin Sonopuls HD60, Germany). Subsequently, debris was removed by centrifugation at 16000 *g* for 10 min. The crude cell extract was used to determine β-galactosidase activity and protein concentration. For PCN estimation, an appropriate volume of sample was taken depending on the densities of the cultures (OD_600_) to ensure sufficient biomass for DNA isolation. Cells were pelleted by centrifugation and stored at −80°C until further use.

### β-Galactosidase assay

β-Galactosidase activity was determined using *o*-nitrophenyl-β-D-galactopyranoside (*o*NPG) as the substrate as described previously [19]. In brief, the assay was performed at an *o*NPG concentration of 22 mM *o*NPG, pH 6.5, and 30°C. One unit of *o*NPG activity is defined as the amount of enzyme releasing 1 μmol of *o*NP per minute under these conditions. Protein concentration was determined by the method of Bradford using bovine serum albumin as the standard.

### SDS-PAGE analysis

The cell-free crude extracts were analysed by SDS-PAGE following the previous protocol [29]. The protein bands were stained with Coomassie Brilliant Blue G250 (Sigma, Switzerland).

### DNA isolation and purification for measurement of plasmid copy number (PCN)

DNA from bacterial cells was isolated and purified using the phenol-chloroform extraction method as described previously [36]. Purified bacterial DNA was stored at −2°C until further use.

### Quantitative reverse transcriptase PCR (qPCR)

#### Oligonucleotide primers

The erythromycin resistance gene *ermB* and the *16S rRNA* gene were chosen as representatives for plasmid DNA and genomic DNA, respectively. The oligonucleotides Ery^R^-f, Ery^R^-r, 16 s-f and 16 s-r (Table 1) were used for qPCR. All primers were obtained from VBC-Biotech (Vienna, Austria).

**Table 1:** **equences of the primers used for qPCR**

**Target gene**	**Primers**	**Sequence (5′ → 3′)**	**Reference**
Plasmid DNA (Erythromycin resistant gene)	Ery^R^_f	CCGTGCGTCTGACATCTATC	This study
	Ery^R^_r	TGCTGAATCGAGACTTGAGTG	
Genomic DNA (16S-rRNA)	16s_f	TGATCCTGGCTCAGGACGAA	[42]
	16s_r	TGCAAGCACCAATCAATACCA	

f denotes forward primers.

r denote reverse primers.

#### qPCR using SYBR Green I

The thermal cycling system iCycler together with the myIQ single Color Real-Time PCR Detection system (Biorad, CA, USA) were used for qPCR amplification and detection. The qPCR reactions were carried out in duplicates of 25-μl reaction mixtures in 96-well plates (iCycler, Biorad) sealed with optical adhesive covers (Microseal ‘B’ film, Biorad). Each reaction contained 250 nM of each primer, 12.5 μl of Perfecta SYBR Green Super mix of IQ (Quanta Biosciences, MD, USA) and 2.5 μl of template DNA (about 50 ng). Negative controls prepared by replacing template DNA with diethylpyrocarbonate (DEPC)-treated water, were included in each run to ensure the absence of DNA contaminants in the reagents. The concentration of primers, annealing temperature and template DNA concentrations had been optimized before the actual experiments as previously described [37]. The qPCR reactions were conducted as follows: initial denaturation at 95°C for 3 min followed by 50 cycles of 20 s at 95°C, 20 s at 60°C, and 72°C for 10 s. Fluorescence was measured at the end of each extension step at 72°C. The temperature was increased from 55°C to 95°C at a rate of 0.2°C per s to establish the melting curve. The threshold cycle values (C_t_) were automatically determined by MyIQ Optical System software (version 2.0) (Biorad).

#### Calculation of the PCN value

Based on PCN definition, which is the number of copies of a plasmid present per chromosome in bacteria [38,39], the PCN can be calculated using equation (1) as previously reported [40]:

1 $$ PCN=\frac{{E_c}^{C_{tc}}}{{E_p}^{C_{tp}}} $$

where *E*_*c*_*, C*_*tc*_ and *E*_*p*_*, C*_*tp*_ are the amplification efficiencies and the threshold cycle values of the amplicon representing chromosome and plasmid, respectively. The equivalence between the amplification efficiency (*E*) of plasmid and chromosomal amplicons was confirmed in validation experiments as described previously [41].

#### Validation of the reverse transcriptase PCR reaction

A series of 10-fold dilutions of template DNA was used to run reverse transcriptase PCR reactions in order to estimate C_t_ values and to subsequently calculate the ∆C_t_ values for the two primer pairs. The amplification efficiencies for the 16S and Ery^R^ primer sets calculated based on the slope of the regression lines of the plots of C_t_ versus the logarithm of DNA dilution were found to be equivalent, i.e., 0.96 and 0.97, respectively. This is also corroborated by the plot of ∆C_t_ versus log_10_ (DNA dilution), where a regression line with a slope of 0.04 was obtained. This indicates that the ∆∆C_t_ method can be used in this study for the two primers sets, 16S and Ery^R^ [41].

### High-performance liquid chromatography

Glucose and lactic acid in fermentation samples were analyzed by high performance liquid chromatography (HPLC) using a Dionex system (Sunnyvale, CA, USA) equipped with an Aminex HPX87-H column (300 × 7.8 mm) from Biorad and 0.005 M sulphuric acid as eluent at a flow rate of 0.6 ml/min, and separation temperature was at 60°C. Interested components were detected by RID detector.

### Statistical analysis

All experiments and measurements were performed at least in duplicate, and the data are given as the mean ± standard deviation when appropriate. The standard deviation was always less than 5%.

## Abbreviations

IP: Inducing pheromone
PCN: Plasmid copy number
MRS medium: de Man, Rogosa and Sharpe medium
*o*NP: *o*-nitrophenol
*o*NPG: *o*-nitrophenyl-β-D-galactopyranoside
